# Pathological fear, anxiety and negative affect exhibit distinct neurostructural signatures: evidence from psychiatric neuroimaging meta-analysis

**DOI:** 10.1038/s41398-022-02157-9

**Published:** 2022-09-23

**Authors:** Xiqin Liu, Benjamin Klugah-Brown, Ran Zhang, Huafu Chen, Jie Zhang, Benjamin Becker

**Affiliations:** 1 The Center of Psychosomatic Medicine, Sichuan Provincial Center for Mental Health, Sichuan Provincial People’s Hospital, MOE Key Laboratory for Neuroinformation, University of Electronic Science and Technology of China, 611731 Chengdu, P. R. China; 2 Institute of Science and Technology for Brain Inspired Intelligence, Fudan University, 200433 Shanghai, P. R. China; 3 Key Laboratory of Computational Neuroscience and Brain Inspired Intelligence, Fudan University, Ministry of Education, 200433 Shanghai, P. R. China

**Keywords:** Psychiatric disorders, Human behaviour

## Abstract

Internalizing disorders encompass anxiety, fear and depressive disorders, which exhibit overlap at both conceptual and symptom levels. Given that a neurobiological evaluation is lacking, we conducted a Seed-based D-Mapping comparative meta-analysis including coordinates as well as original statistical maps to determine common and disorder-specific gray matter volume alterations in generalized anxiety disorder (GAD), fear-related anxiety disorders (FAD, i.e., social anxiety disorder, specific phobias, panic disorder) and major depressive disorder (MDD). Results showed that GAD exhibited disorder-specific altered volumes relative to FAD including decreased volumes in left insula and lateral/medial prefrontal cortex as well as increased right putamen volume. Both GAD and MDD showed decreased prefrontal volumes compared to controls and FAD. While FAD showed less robust alterations in lingual gyrus compared to controls, this group presented intact frontal integrity. No shared structural abnormalities were found. Our study is the first to provide meta-analytic evidence for distinct neuroanatomical abnormalities underlying the pathophysiology of anxiety-, fear-related and depressive disorders. These findings may have implications for determining promising target regions for disorder-specific neuromodulation interventions (e.g. transcranial magnetic stimulation or neurofeedback).

## Introduction

Anxiety disorders (AD) constitute the most prevalent diagnostic group of mental disorder and cause considerable suffering, disability and economic costs [1]. AD comprise a group of heterogeneous disorders that share features of excessive fear and anxiety [2]. Recent overarching conceptualizations based on the DSM-5 propose that AD can be placed along a fear–anxiety continuum ranging from excessive fear-based responses to imminent specific threats in fear-related anxiety disorders (FAD, e.g., social anxiety disorder, SAD; specific phobias, SP; panic disorder, PD; and agoraphobia, AG) to a rather diffuse anxious apprehension of events in anxiety-related anxiety disorders such as generalized anxiety disorder (GAD) [3]. This echoes findings on the psychopathological factor model in internalizing disorders indicating that SAD, SP, PD and AG originate from the higher-order “fear” dimension, whereas GAD and major depressive disorder (MDD) originate from the “anxious-misery” or “distress” dimension [4–6]. On the other hand, subcategories of AD are often highly co-morbid with each other as well as with other emotional (internalizing) disorders [7]. Particularly, GAD and MDD exhibit symptomatic overlap (e.g., negative affect, worry) [8] and common genetic factors [9]. Despite ongoing debates about the nosology of psychiatric disorders and overarching symptom domains [10], the neurobiological substrates underlying these conceptualizations remain unclear.

Animal models and human neuroimaging studies have demonstrated that anxiety and fear are regulated by distinct neurobiological circuits such that the fear response is mediated by the central nucleus of the amygdala (CeA), and anxiety is mediated by the bed nucleus of the stria terminalis (BNST) [11, 12]. While the segregation has been translated into the Acute Threat (fear) and Potential Threat (anxiety) domains proposed in the Research Domain Criteria (RDoC) framework [13], accumulating evidence from neuroimaging studies in healthy individuals suggests a shared neurofunctional basis of anxiety, fear and general negative affect [14–16]. In contrast, research on pathological anxiety (i.e., GAD) and pathological fear generated inconsistent results [17] with respect to a shared neurobiological basis which may be due to small sample sizes as well as clinical and analytic variability in the original studies [18]. Recent mega-analyses from ENIGMA working groups synergize imaging data across multiple sites worldwide to generate more robust and replicable findings on structural alterations in anxiety and affective disorders [19–21]. These mega-analyses did not reveal significant main effect of diagnosis for GAD on brain structure including cortical thickness, cortical surface area and subcortical volume [22] or for SAD on gray matter volume (GMV) in whole-brain voxel-based morphometry (VBM) analysis [23].

Another effort to address the issues of original studies is to conduct quantitative neuroimaging meta-analyses by pooling data across multiple studies. Several previous meta-analyses have examined neurostructural alterations within diagnostic entities [24–26] or overarching disorder categories including AD and MDD [27, 28]. For instance, decreased GMV in the medial prefrontal cortex (mPFC), superior temporal gyrus (STG) and insula have been concurrently found in separate meta-analyses in GAD [24], FAD [29] and MDD [30]. Two recent transdiagnostic meta-analyses have reported GMV deficits in the left inferior frontal gyrus (IFG) in mixed GAD and FAD group [28, 31], whereas the cerebellum and the STG has been found to differentiate the MDD from the AD group [28]. Although these findings provide indirect evidence for common and separable brain alterations in internalizing disorders based on rather lenient statistical thresholds, the differentiation along the fear vs anxiety spectrum rooted in basic neuroscience and diagnostic differentiations has not been examined.

Against the background of overarching conceptual frameworks as well as translational animal models suggesting a shared and separable neurobiological basis of fear, anxiety and general negative affect, we conducted preregistered comparative meta-analyses including coordinates as well as original maps of case-control VBM studies in GAD, FAD (including SAD, SP, AG and PD) and MDD using the recently developed Signed Differential Mapping with Permutation of Subject Images (SDM-PSI, https://www.sdmproject.com/) approach [32]. Notably, our meta-analysis focused on VBM studies given that the number of whole-brain studies using other morphometric measurements would currently not allow a robust meta-analytic computation [e.g. suitable surface-based morphometry (SBM) studies for GAD would be limited to Andreescu et al. [33], Molent et al. [34], Strawn et al. [35]]. Based on the previous literature, we hypothesized decreased GMV in GAD and MDD in prefrontal regions engaged in cognitive processes and emotional regulation, such as the IFG in GAD [24] or the orbitofrontal cortex (OFC) in MDD [30], while FAD patients were expected to exhibit GMV differences in regions engaged in emotion generation such as visual processing regions [36] and limbic regions such as the amygdala [37]. Moreover, we expected common GMV reductions across disorders in regions engaged in the representation of negative affect such as the insula [27, 38].

## Methods

### Search and study selection

**Fig. 1:**
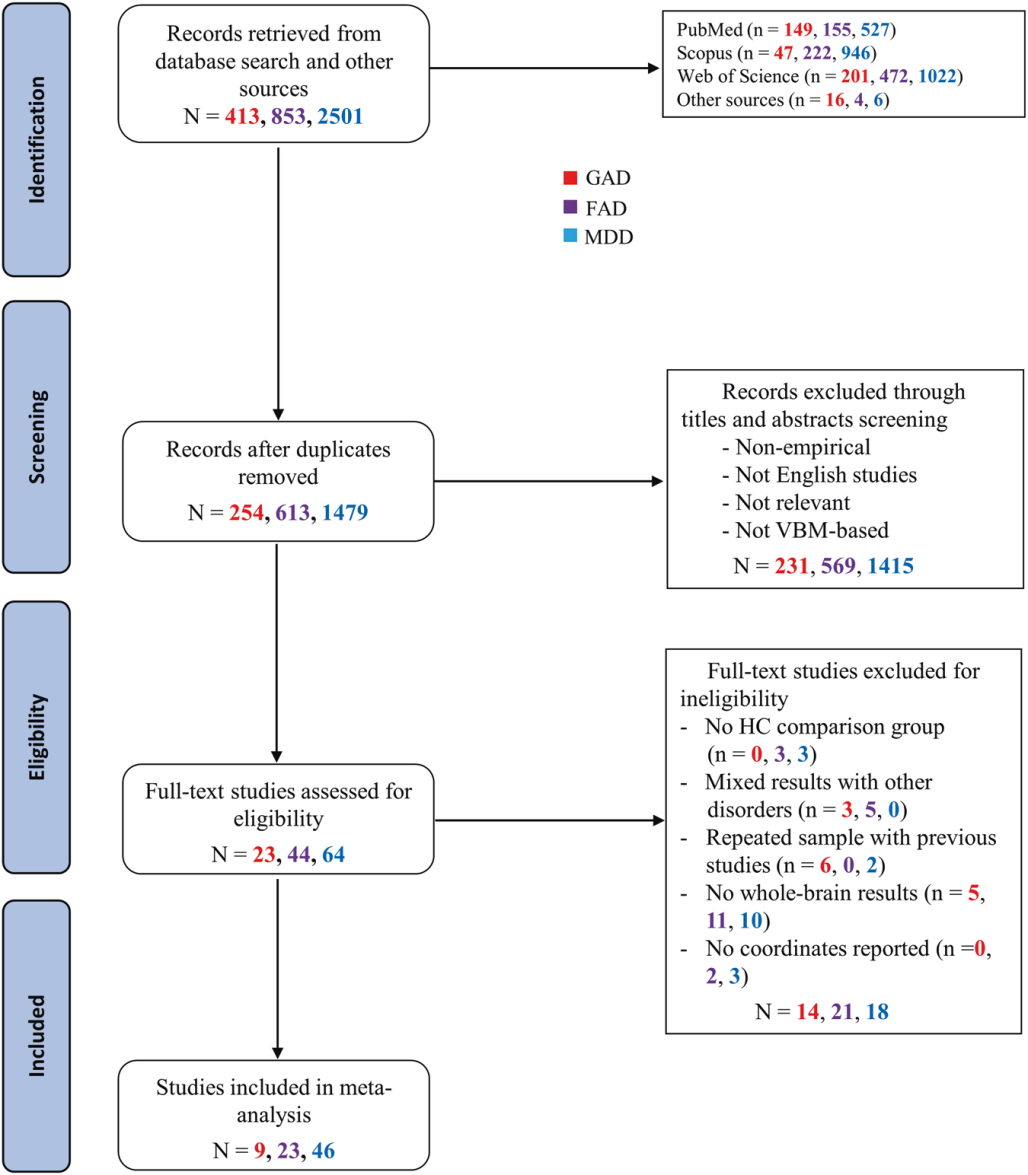
PRISMA flow diagram of study selection in the current meta-analysis. FAD fear-related anxiety disorder, GAD generalized anxiety disorder, HC healthy controls, MDD major depressive disorder, VBM voxel-based morphometry. Note: The Arabic numerals in the figure represent the number of studies in each step.

A comprehensive literature search was conducted in PubMed, Web of Knowledge, and Scopus databases for case-control VBM studies comparing GAD, or SAD/SP/AG/PD, or MDD patients with healthy controls (HC) through December 20, 2020 (Fig. 1), according to the PRISMA guidelines [39]. Search terms are provided in Supplementary Methods.

Key Inclusion criteria: (1) whole-brain VBM comparisons reported between patients of GAD, SAD/SP/AG/PD or MDD and HC and (2) coordinates provided in Talairach or Montreal Neurological Institute (MNI) space (detailed inclusion criteria see Supplementary Methods). In case of overlapping samples between studies, only the record with the greatest sample size was included. For studies using longitudinal treatment designs, only pre-treatment (baseline) data were included. Studies of treatment-resistant patients and remitted depression were excluded to reduce the pathophysiological heterogeneity within the diagnostic groups [40]. To match illness duration between patient groups, studies of first-episode MDD with a mean illness duration ≤ 2 years were excluded. Original whole-brain *t*-maps and missing data were requested from authors via e-mail.

X.Q.L and R.Z. independently screened and assessed all articles achieving 100% agreement. Peak coordinates and effect sizes of significant GMV alterations in both directions (i.e., patient > HC, and patient < HC) and other basic information (e.g., sample size, age, sex, etc.) were independently extracted by X.Q.L and R.Z.

### Meta-analysis

Voxel-wise meta-analyses were performed using Seed-based d Mapping as implemented in the most recent SDM-PSI (version 6.21, https://www.sdmproject.com/) [32]. Details of this method are provided elsewhere [32, 41]. In summary, all meta-analyses were conducted using peak coordinates including their effective sizes (*t*-values) or original whole-brain t maps of individual studies if provided. The unbiased maximum likelihood estimation (MLE) was used to create whole-brain effect size and variance map based on the MetaNSUE algorithm. SDM-PSI further allows family-wise error (FWE) correction for multiple comparisons with common permutation tests using threshold-free cluster enhancement (TFCE) thus increasing reliability. To prevent a single study or few studies from driving the results, SDM-PSI uses a leave-one-out jackknife procedure.

We performed a three-step meta-analytic approach to determine common and disorder-specific brain structural alterations between anxiety-related, fear-related and depressive disorders (similar approach see also [42–44]): (1) to characterize robust GMV deficits for each disorder in comparison to their respective HC, we initially conducted separate meta-analyses for each of the disorder groups (i.e., GAD vs HC, FAD vs HC, MDD vs HC). Subgroup meta-analyses in FAD were further performed to account for heterogeneity between specific diagnostic subgroups within this category (SAD, SP and PD; no AG studies were identified); (2) to assess disorder-specific GMV abnormalities, we computed a quantitative contrast analyses that compared the disorder groups (i.e., GAD vs FAD, GAD vs MDD, FAD vs MDD, relative to their respective HCs) by calculating the difference in each voxel covarying for age and sex, and using standard randomization tests to establish statistical significance; (3) to examine shared GMV abnormalities across the three disorder groups (relative to the respective HCs), a multimodal conjunction analysis was conducted by accounting for error in the estimation of *p*-values within each voxel from the separate meta-analytic maps. A TFCE-based FWE corrected threshold *p* < 0.05 with a voxel extent ≥ 10 was initially used throughout analyses. Further, a more liberal threshold balancing between Type I and Type II errors (uncorrected *p* < 0.0025 and voxel extent ≥ 10 voxels) was explored in separate meta-analyses in line with previous studies [45], and in conjunction analyses as suggested in bimodal tests [46]. We increased the sensitivity of the analyses by combining peak coordinates with raw statistical maps obtained from some of the original studies.

To examine a potential confounding influence of demographic and clinical variables, meta-regression analyses were performed within each patient group (in case variables were reported in *≥* 9 studies, as recommended by Radua et al. [47]). These analyses examined whether the volume of the identified regions from the separate meta-analyses was associated with age, female ratio, illness duration, percentage of medication, percentage of comorbidity and symptom severity, in line with previous studies [29, 47, 48]. TFCE-based FWE corrected threshold (*p* < 0.05, voxel extent ≥10) was used. To further control for potential confounding effects of comorbidity on the identification of shared and distinct structural alterations, we here included further statistical strategies to carefully control for comorbidity effects in the comparative and conjunctive meta-analyses (detailed description and results see Supplementary Methods, Supplementary Table S2 and Fig. S1).

Heterogeneity analyses with *I*^2^ statistics were carried out to statistically evaluate the inter-study heterogeneity of individual clusters identified from the meta-analyses [47, 49], in which a value of 0% to 30% indicates mild heterogeneity and >50% indicates substantial heterogeneity [50]. Publication bias was assessed with Egger’s test. Additionally, a transdiagnostic meta-analysis pooling the VBM studies of GAD, FAD and MDD was conducted to compare healthy controls with pooled patients for identifying transdiagnostic convergence in structural abnormalities across internalizing disorders. This additional analysis served to increase comparability with previous meta-analytic work on transdiagnostic brain alterations that used the corresponding pooled-across-diagnoses approach [27, 38]. The results of this analysis were thresholded at TFCE-based FWE corrected *p* < 0.05 (see Supplementary Methods and Supplementary Fig. S2).

The meta-analytic protocols were pre-registered on the Open Science Framework (https://osf.io/es2vm). Coordinates and *t*-value files are available at https://osf.io/46uc2/. Unthresholded whole-brain maps are provided at https://neurovault.org/collections/11343/.

## Results

### Included studies and sample characteristics

Included were 9 GAD studies [35, 51–58], 23 FAD (10 SAD, 11 PD, 2 SP and 0 AG) studies [23, 36, 59–79], and 46 MDD studies [77, 80–126]. Whole-brain *t*-maps were available for 1 GAD study [51], 2 FAD (SAD) studies [23, 74] and 1 MDD study [122]. Tables 1 to 3 provide demographic and clinical information of the individual studies, summarizing sample sizes (226 GAD patients vs 226 HC, 918 FAD patients vs 989 HC, 2,575 MDD patients vs 2,866 HC), female ratio and age for each disorder group and providing details on the sex and age differences between patients and HC within each disorder group, respectively.

**Table 1:** Demographic and clinical characteristics of the 9 GAD VBM datasets included in the meta-analysis (*n* = 226).

Study	Number (female)		Age Mean (SD)		Duration years (SD)	Medication	Comorbidity	Scanner/FWHM (mm)	*p* value	Summary findings
	GAD	HC	GAD	HC						
Chen et al. 2020^a^	72 (41)	57 (30)	39.04 (11.82)	40.91 (15.62)	4.56 (4.86)	Medication load index: 1.67 ± 0.69	0	3 T/8	*p* < 0.05 (GRF)	GAD < HC: L/R sgACC/vmPFC, L ITG, R Insula, R dmPFC
Hilbert et al. 2015	19 (16)	24 (17)	33.47 (8.90)	32.25 (9.33)	NA	Drug naïve	12 MDD, 2 dysthymia, 13 other anxiety disorders	3 T/8	*p* < 0.05 (FWE)	GAD > HC: R Striatum
Kim et al. 2018	16 (6)	16 (6)	33.00 (8.60)	32.50 (7.30)	5.60 (7.50)	Medicated (*n* = 10)	NA	3 T/6	*p* < 0.001 (uncorr)	GAD < HC: R ACC, L Precuneus, L dlPFC, L OFG, L SOG, L Insula, L STG, L mPFC
Liao et al. 2014	26 (13)	25 (12)	16.85 (0.69)	16.72 (0.83)	NA	Drug naïve	0	3 T/8	*p* < 0.05 (FWE)	GAD > HC: R Putamen
Ma et al. 2019	21 (9)	20 (10)	34.92 (9.49)	35.96 (8.71)	2.33 (3.14)	Drug-free (>6 month)	0	3 T/6	*p* < 0.05 (AlphaSim)	GAD < HC: R Precentral gyrus, R SFG
Makovac et al. 2016	19 (16)	19 (16)	30.00 (6.90)	29.20 (9.80)	16.78 (8.01)	Medicated (*n* = 2)	0	1.5 T/8	*p* < 0.05 (FWE)	GAD < HC: R Supramarginal gyrus/postcentral gyrus, L Postcentral gyrus, R Precentral gyrus, L Supramarginal gyrus
Moon et al. 2014	22 (9)	22 (9)	37.00 (10.70)	33.40 (9.70)	4.50 (6.60)	NA	NA	3 T/8	*p* < 0.001 (uncorr)	GAD < HC: L Midbrain, L Thalamus, L Hippocampus, L Insula, L STG
Schienle et al., 2011	16 (16)	15 (15)	22.90 (4.10)	23.70 (3.70)	3.10 (4.70)	Drug naïve	0	3 T/12	*p* < 0.05 (FWE)	–
Strawn et al., 2013	15 (8)	28 (17)	13.00 (2.00)	13.00 (2.00)	NA	Drug naïve	6 ADHD, 6 other anxiety disorders	4 T/8	*p* < 0.001 (uncorr)	GAD > HC: R precentral gyrus, R Precuneus; GAD < HC: R PCC, L OFG
**Total Sample**	226 (134)	226 (132)	31.38 (12.45)^b^	29.97 (13.83)^b^	**Weighted t test**		sex: *t* = 0.02, *p* > 0.05		age: *t* = 0.34, *p* > 0.05	

*ACC* anterior cingulate cortex, *dlPFC* dorsolateral prefrontal cortex, *dmPFC* dorsomedial prefrontal cortex, *FWE* family-wise error, *FWHM* full width at half maximum, *GAD* generalized anxiety disorder, *GR* Gaussian random field, *HC* healthy controls, *ITG* inferior temporal gyrus, *L* left hemisphere, *mPFC* medial prefrontal cortex, *NA* not available, *OFG* orbitofrontal gyrus, *PCC* posterior cingulate cortex, *R* right hemisphere, *SFG* superior frontal gyrus, *sgACC* subgenual anterior cingulate cortex, *SOG* superior occipital gyrus, *STG* superior temporal gyrus, *uncorr* uncorrected, *VBM* voxel-based morphometry, *vmPFC* ventromedial prefrontal cortex.

^a^Studies that provided original whole-brain *t*-maps.

^b^Weighted averages. Note that the p values in the weighted t test have been corrected (Bonferroni) for multiple comparisons.

**Table 2:** Demographic and clinical characteristics of the 23 FAD VBM datasets included in the meta-analysis (*n* = 918).

Study	Disorder type	Number (female)		Age Mean (SD)		Duration years (SD)	Medication	Comorbidity	Scanner/FWHM (mm)	*p* value	Summary findings
		Patients	HC	Patients	HC						
Bas-Hoogendam et al. 2017^a^	SAD	174 (102)	213 (106)	30.60 (10.00)	32.40 (10.50)	15.80 (7.10)	Medicated (n = 24)	8 MDD, 2 MDD + PD, 10 GAD, 3 GAD + SP, 2 GAD + PD, 3PD, 6 SP, 26 unknown	3 T/7.5 mm	*p* < 0.05 (FWE)	–
Cheng et al. 2015	SAD	20 (7)	30 (9)	23.30 (3.70)	26.20 (6.60)	3.99 (3.68)	Drug naïve	0	3 T/8 mm	*p* < 0.05 (FWE)	–
Frick et al. 2014	SAD	48 (24)	29 (16)	33.80 (9.30)	23.70 (2.00)	NA	Drug naïve	10 GAD; 7 SP; 3 MDD; 2 PD	NA/8 mm	*p* < 0.05 (corr)	SAD > HC: L/R Lingual gyrus, L FFG, R Secondary occipital cortex
Irle et al. 2014	SAD	67 (35)	64 (31)	31.00 (10.00)	32.00 (10.00)	15.00 (9.00)	Medicated (*n* = 6)	16 MDD; 7 SP; 5 PD; 1 GAD	3 T/8 mm	*p* < 0.001 (uncorr)	–
Kawaguchi et al. 2016	SAD	13 (8)	18 (0)	36.20 (11.80)	33.80 (9.60)	23.30 (14.40)	NA	4 MDD; 1 PD	3 T/8 mm	*p* < 0.05 (FWE)	–
Liao et al. 2011	SAD	18 (6)	18 (5)	22.70 (3.80)	21.90 (3.70)	4.10 (3.35)	Drug naïve	0	3 T/8 mm	*p* < 0.05 (AlphaSim)	SAD > HC: R mPFC; SAD < HC: L PHG, R ITG
Meng et al. 2013	SAD	20 (6)	19 (6)	21.80 (3.70)	21.60 (3.70)	4.21 (3.82)	Drug naïve	0	3 T/12 mm	*p* < 0.05 (AlphaSim)	SAD < HC: L/R Thalamus, R Amygdala, R Precuneus
Talati et al. 2013	SAD	33 (9)	37 (19)	31.50 (8.20)	31.40 (9.10)	NA	Medicated (*n* = 9)	11 MDD;5 GAD; 4 SP, 1 OCD, 1 DUD, 2 AUD	1.5 T/8 mm	*p* < 0.05 (nonstationary corr)	SAD > HC: R supramarginal and angular cortices; SAD < HC: L temporal pole
Tükel et al. 2015^a^	SAD	27 (15)	27 (15)	27.70 (6.70)	27.70 (5.80)	13.80 (7.00)	Drug naïve	0	1.5 T/8 mm	*p* < 0.05 (AlphaSim)	SAD > HC: L Superior parietal and precuneus, R middle and inferior temporal areas, R FFG
Zhao et al. 2017	SAD	24 (9)	41 (15)	24.50 (4.00)	27.10 (7.20)	7.60 (3.80)	Drug naïve	0	3 T/8 mm	*p* < 0.001 (FDR)	SAD < HC: L/R Putamen, L/R OFC, L/R Thalamus
**Total Subsample**	**SAD**	444 (221)	496 (222)	29.69 (9.18)^b^	29.95 (9.58)^b^	**Weighted t test**		sex: *t* = −1.03, *p* > 0.05		age: *t* = 1.70, *p* > 0.05	
Asami et al. 2009	PD	24 (15)	24 (15)	37.03 (9.98)	37.01 (8.70)	3.90 (3.43)	Medicated (*n* = 36)	13 AG, 3 MDD, 1 dysthymia	1.5 T/12 mm	*p* < 0.05 (FDR)	PD < HC: L/R dmPFC, R vmPFC, R Amygdala, R ACC, L/R STG, L/R Insula, L/R Lateral occipitotemporal gyrus, L cerebellar vermis
Kunas et al. 2020	PD	143 (89)	178 (101)	33.65 (11.00)	31.63 (10.35)	NA	Drug naïve	51 MDD	3 T/8 mm	*p* < 0.001 (uncorr)	PD < HC: L MTG
Lai & Wu, 2012	PD	30 (19)	21 (11)	47.03 (10.63)	41.14 (11.81)	NA	Drug naïve	13 AG	3 T/7.5 mm	*p* < 0.005 (FWE)	PD < HC: L OFC, L IFG, R Insula, L STG
Lai & Wu, 2015	PD	53 (28)	54 (29)	43.28 (10.11)	40.38 (10.51)	5.35 (2.37)	Drug naïve	0	3 T/7.5 mm	*p* < 0.05 (FWE)	PD < HC: R IFG R Insula
Massana et al. 2003	PD	18 (11)	18 (10)	36.80 (11.30)	36.70 (8.80)	NA	Drug naïve	15 AG	1.5 T/12 mm	*p* < 0.05 (corr)	PD < HC: L PHG
Na et al. 2013	PD	22 (9)	22 (11)	40.18 (10.54)	40.18 (12.38)	NA	Drug naïve	12 AG	3 T/8 mm	*p* < 0.05 (FWE)	PD < HC: L/R SOG, cuneus
Protopopescu et al. 2006	PD	10 (6)	23 (11)	33.50 (9.70)	28.70 (7.50)	NA	Medicated (*n* = 1)	2 AG	3 T/12 mm	*p* < 0.05 (GRF)	PD > HC: Brainstem
Sobanski et al. 2010	PD	17 (9)	17 (9)	34.90 (6.70)	33.10 (6.20)	7.90 (5.20)	NA	16 AG, 1 SAD, 1 GAD, 2 AD, 2 PPD	1.5 T/12 mm	*p* < 0.05 (FWE)	PD < HC: R MTG, R OFC
Talati et al. 2013	PD	16 (13)	20 (9)	31.80 (10.00)	31.40 (6.70)	13.40 (6.60)	Medicated (*n* = 9)	3 MDD, 2 GAD,5 SP, 2 OCD,4 DUD,4 AUD	1.5 T/8 mm	*p* < 0.05 (corr)	PD > HC: L/R Cuneus, lingual; PD < HC: R precentral, postcentral, R middle cingulate
Uchida et al. 2008	PD	19 (16)	20 (16)	37.05 (9.75)	36.45 (9.93)	8.34 (6.01)	Medicated (*n* = 15)	14 AG, 3 MDD, 2 dysthymia	1.5 T/12 mm	*p* < 0.001 (uncorr)	PD > HC: L Insula and STG, L Midbrain and pons; PD < HC: R ACC
Yoo et al. 2005	PD	18 (9)	18 (7)	33.30 (7.10)	32.00 (5.80)	3.60 (2.20)	Medicated (*n* = 10)	0	3 T/8 mm	*p* < 0.05 (corr)	PD < HC: L/R Putamen, R Precuneus gyrus, R ITG, L STG, L SFG
**Total Subsample**	**PD**	370 (224)	415 (229)	37.01 (11.17)^b^	34.50 (10.45)^b^	**Weighted t test**		sex: *t* = −0.54, *p* > 0.05		age: *t* = 25.3, *p* < 0.001	
Hilbert et al. 2015	SP	59 (45)	37 (28)	23.95 (4.90)	22.76 (3.88)	NA	Drug naïve	0	1.5 T/12 mm	*p* < 0.05 (FWE)	Specific phobia > HC: R ACC, R Calcarine sulcus, R FFG, L medial OFC, L Precuneus, R Vermis
Schienle et al. 2013	SP	45 (25)	41 (23)	30.39 (10.61)	29.24 (9.00)	16.69 (10.74)	Drug naïve	0	3 T/8 mm	*p* < 0.001 (uncorr)	–
**Total Subsample**	**SP**	104 (70)	78 (51)	26.74 (8.48)^b^	26.17 (7.73)^b^						
**Total Sample**	**FAD**	918 (515)	989 (502)	32.30 (10.71)^b^	31.56 (10.18)^b^	**Weighted t test**		sex: *t* = 0.29, *p* > 0.05		age: *t* = 0.55, *p* > 0.05	

*ACC* anterior cingulate cortex, *AD* adjustment disorder with mixed disturbance of emotions and conduct, *AG* agoraphobia, *AUD* alcohol use disorder, *corr* corrected, *dlPFC* dorsolateral prefrontal cortex, *dmPFC* dorsomedial prefrontal cortex, *DUD* drug use disorder, *FAD* fear-related anxiety disorder, *FDR* false discovery rate, *FFG* fusiform gyrus, *FWE* family-wise error, *FWHM* full width at half maximum, *GRF* Gaussian random field, *HC* healthy controls, *IFG* inferior frontal gyrus, *IOG* inferior occipital gyrus, *IPL* inferior parietal lobule, *ITG* inferior temporal gyrus, *L* left hemisphere, *LOC* lateral occipital cortex, *MCC* medial cingulate cortex, *MDD* major depressive disorder, *MFG* medial frontal gyrus, *MOG* middle occipital gyrus, *mPFC* medial prefrontal cortex, *MTG*, middle temporal gyrus, *NA* not available, *OCD* obsessive-compulsive disorder, *OFC* orbitofrontal cortex, *PCC* posterior cingulate cortex, *PCG* paracingulate gyrus, *PD* panic disorder, *PHG* parahippocampal gyrus, *PPD* paranoid personality disorder, *PTSD* post-traumatic stress disorder, *R* right hemisphere, *SAD* social anxiety disorder, *SFG* superior frontal gyrus, *SMA* supplementary motor area, *SOG* superior occipital gyrus, *SP* specific phobia, *STG* superior temporal gyrus, *TOFC* temporal-occipital fusiform cortex, *uncorr* uncorrected,*VBM* voxel-based morphometry, *vmPFC* ventromedial prefrontal cortex.

^a^Studies that provided original whole-brain *t*-maps.

^b^Weighted averages. Note that the p values in the weighted t test have been corrected (Bonferroni) for multiple comparisons.

**Table 3:** Demographic and clinical characteristics of the 46 MDD VBM datasets included in the meta-analysis (*n* = 2,575).

Study	Number (female)		Age Mean (SD)		Duration years (SD)	Medication	Comorbidity	Scanner/FWHM (mm)	*p* value	Summary findings
	MDD	HC	MDD	HC						
Abe et al. 2010	21 (10)	42 (20)	48.10 (13.50)	48.00 (13.20)	6.00 (7.2)	Medicated (*n* = 19)	0	1.5 T/6 mm	*p* < 0.05 (FDR)	MDD < HC: R PHG, L/R Middle frontal gyrus, L IOG, L SMA, R STG, L Parietal lobe, L/R ACC
Ahn et al. 2016	34 (29)	26 (19)	32.43 (7.76)	31.40 (7.60)	NA	NA	NA	3 T/8 mm	*p* < 0.05 (AlphaSim)	MDD > HC: L Postcentral gyrus, R Postcentral gyrus, L/R Parieto-occipital cortex, L/R Putamen, R Thalamus, L/R Hippocampus, L/R Cerebellum; MDD < HC: L/R OFC, R dmPFC, R dorsal ACC, L/R MOG, L Cuneus
Amico et al. 2011	33 (14)	64 (28)	32.00 (8.00)	30.40 (9.30)	3.40 (5.00)	Medicated (n = 27)	0	1.5 T/8 mm	*p* < 0.05 (FWE)	–
Arnone et al. 2013	39 (27)	66 (46)	36.30 (8.80)	32.10 (9.30)	14.30 (8.10)	Drug naïve	0	1.5 T/8 mm	*p* < 0.05 (FWE)	MDD < HC: L/R Hippocampus/PHG, L/R FFG/ITG, L/R Ventral striatum
Bergouignan et al. 2009	20 (17)	21 (14)	33.16 (9.58)	28.21 (5.50)	8.45 (9.03)	Medicated (*n* = 20)	0	1.5 T/8 mm	*p* < 0.05 (FDR)	MDD < HC: R Cingulate gyrus, R MTG, R Posterior lobe, R SPL, R PHG, L Inferior semi-lunar lobule
Biedermann et al., 2015	46 (34)	35 (22)	50.80 (14.90)	46.40 (12.20)	NA	Medicated (*n* = 24)	NA	1.5 T/8 mm	*p* < 0.05 (FWE)	–
Cai et al., 2015	23 (10)	23 (10)	30.00 (7.29)	30.00 (7.29)	4.35 (2.38)	NA	0	3 T/8 mm	*p* < 0.001 (uncorr)	MDD < HC: IFG
Chaney et al. 2014	37 (21)	46 (28)	40.22 (10.02)	36.61 (11.89)	9.65 (10.01)	Medicated (*n* = 24)	0	3 T/10 mm	*p* < 0.05 (FWE)	–
Chen et al. 2016^a^	27 (14)	28 (14)	33.00 (10.80)	33.00 (11.70)	6.58 (7.17)	Drug naïve	0	3 T/6 mm	*p* < 0.05 (AlphaSim)	MDD > HC: R Postcentral gyrus
Chen et al. 2020	22 (18)	22 (18)	28.70 (13.30)	27.40 (10.20)	4.40	Medicated (*n* = 17)	0	3 T/8 mm	*p* < 0.001 (uncorr)	MDD < HC: R Occipital fusiform gyrus, L Postcentral gyrus
Dannlowski et al. 2015	171 (105)	512 (289)	38.60 (11.70)	33.30 (11.60)	NA	Medicated (*n* = 171)	0	3 T/8 mm	*p* < 0.001 (Monte Carlo)	MDD < HC: R PHG/HC/amygdala, R Insula/putamen, IFG, L Lingual gyrus, FFG, Cerebellum, IOG, L/R MCC, L STG/MTG, L/R Thalamus
Fang et al. 2015	20 (8)	18 (8)	59.20 (3.70)	59.10 (7.50)	3.60 (1.10)	NA	NA	1.5 T/8 mm	*p* < 0.05 (corrected)	MDD < HC: L/R ITG, L IFG, L Precuneus, R MCC, R FFG, R Cuneus
Förster et al. 2020	63 (33)	46 (22)	42.43 (11.91)	45.35 (8.37)	NA	NA	19 anxiety disorders	3 T/8 mm	*p* < 0.05 (FWE)	–
Grieve et al. 2013	102 (54)	34 (16)	33.80 (13.10)	31.50 (12.40)	11.30 (11.80)	NA	0	3 T/8 mm	*p* < 0.05 (FDR)	MDD < HC: R Rectal gyrus, R IFG, L FFG, R ITG, L MTG,
Hagan et al. 2015	109 (81)	36 (26)	15.56 (1.27)	15.65 (1.45)	NA	Medicated (*n* = 37)	0	3 T/7 mm	*p* < 0.05 (FWE)	–
Inkster et al. 2011	145 (94)	183 (110)	49.45 (13.17)	48.00 (13.30)	14.30 (10.65)	NA	0	1.5 T/10 mm	*p* < 0.05 (FWE)	MDD < HC: R SPL
Kandilarova et al. 2019	39 (29)	42 (29)	47.70 (13.90)	42.60 (13.70)	10.80 (8.89)	Medicated (*n* = 37)	0	3 T/8 mm	*p* < 0.05 (FDR)	MDD < HC: L MFG/ACC
Klauser et al. 2015	56 (40)	33 (21)	34.02 (8.96)	34.71 (9.93)	10.29 (8.08)	Medicated (*n* = 33)	0	1.5 T/10 mm	*p* < 0.05 (permutation)	MDD < HC: L/R vmPFC
Lee et al. 2011	47 (42)	51 (45)	46.00 (9.10)	45.70 (8.00)	3.89 (6.33)	Medicated (*n* = 27)	0	1.5 T/10 mm	*p* < 0.05 (FDR)	MDD < HC: L/R Midbrain, L/R sgACC, L/R Thalamus, L Short insular gyrus, L/R Nucleus accumbens, L/R Amygdala, L/R Hippocampus, L/R FFG, L Lingual gyrus, L/R MTG, R STG, L/R Cerebellum
Lee et al. 2020	20 (20)	21 (21)	42.50 (13.95)	42.30 (10.2)	9.50 (11.33)	Medicated (*n* = 20)	0	1.5 T/10 mm	*p* < 0.05 (FDR)	MDD < HC: L/R Middle frontal gyrus, L/R Rectal gyrus, L/R Short and long insula gyrus, L/R ACC, MCC, L/R PCC, L/R Thalamus, L/R Hypothalamus, L/R Amygdala, L/R Hippocampus, L/R PHG, L Lingual gyrus, L/R STG, MTG, ITG
Leung et al. 2009	17 (17)	17 (17)	45.50 (8.50)	45.80 (9.80)	7.00 (4.09)	Medicated (*n* = 17)	NA	1.5 T/12 mm	*p* < 0.001 (uncorr)	MDD < HC: R ACC, R STG, R SFG, L/R MFG, R FFG, R IFG, L/R Precentral gyrus, L MCC, L Insula, L Angular gyrus, L Precuneus, L MTG, L MCC, PCC
Lu et al. 2018	76 (44)	86 (43)	34.27 (8.37)	33.35 (7.62)	3.74 (4.49)	Drug naïve	0	3 T/8 mm	*p* < 0.05 (GRF)	MDD > HC: L L Caudate, R ITG, L Cerebellum, L Lingual gyrus; MDD < HC: L/R PHG/Hippocampus, R MTG
Lu et al. 2019	30 (13)	48 (30)	23.98 (5.25)	21.50 (3.84)	2.56 (2.19)	Drug naïve	0	3 T/8 mm	*p* < 0.05 (GRF)	MDD > HC: R MOG; MDD < HC: L SPL
Mak et al. 2009	17 (17)	17 (17)	45.50 (8.50)	45.80 (9.80)	NA	Medicated (*n* = 17)	0	1.5 T/8 mm	*p* < 0.001 (uncorr)	MDD < HC: R ACC, R SMA, R Precentral gyrus, R Temporal pole, L Angular gyrus, L Precuneus
Modinos et al. 2014	23 (20)	46 (14)	44.60 (5.50)	25.30 (4.30)	NA	NA	NA	1.5 T/8 mm	*p* < 0.05 (FWE)	MDD < HC: L/R Orbital gyrus, R MFG, L ACC, L/R IPL, R MTG, L MFG
Nakano et al. 2014	36 (22)	54 (27)	49.00 (11.40)	45.40 (16.10)	5.56 (6.74)	Medicated (*n* = 31)	0	1.5 T/8 mm	*p* < 0.05 (FWE)	MDD < HC: R mPFC, R superior OFC
Opel et al. 2019	506 (308)	358 (178)	49.14 (7.28)	52.57 (7.94)	6.73 (7.94)	Medicated (*n* = 441)	Comorbidity index: 1.65 ± 0.81	3 T/8 mm	*p* < 0.05 (FWE)	MDD < HC: R Insula/STG/MTG, L SFG/MFG, R MFG/IFG
Pannekoek et al. 2014	26 (23)	26 (23)	15.40 (1.50)	14.70 (1.50)	NA	Drug naïve	18 anxiety disorder; 5 ADHD	3 T/7 mm	*p* < 0.05 (permutation)	–
Qiu et al. 2016	12 (8)	15 (10)	34.40 (10.10)	33.70 (9.90)	NA	Drug naïve	0	3 T/8 mm	*p* < 0.001 (uncorr)	MDD < HC: R Cingulate gyrus
Redlich et al. 2014	58 (36)	58 (37)	37.60 (10.80)	37.70 (9.70)	10.98 (8.94)	Medicated (*n* *=* 52)	35 anxiety disorder	3 T/8 mm	*p* < 0.05 (AlphaSim)	MDD < HC: L/R Hippocampus, FFG, Lingual gyrus, L Supramarginal gyrus, IPL, L/R MCC, ACC, mPFC, SMA, R Middle frontal gyrus, SFG, L/R Precunues, L MTG/STG, L Caudate
Redlich et al. 2018	20 (15)	21 (12)	16.00 (1.03)	16.57 (1.08)	2.63 (1.70)	Medicated (*n* = 8)	0	3 T/6 mm	*p* < 0.05 (FWE)	–
Rodríguez-Cano et al. 2014	32 (20)	64 (38)	48.68 (12.98)	46.03 (9.83)	10.94 (10.54)	Medicated (*n* = 28)	2 AUD; 2 AG; 1 AD; 1 anxiety disorder; 1 dysthymia	1.5 T/4 mm	*p* < 0.05 (FWE)	MDD < HC: L FFG
Salvadore et al. 2011	58 (37)	107 (60)	38.80 (11.10)	36.20 (10.30)	18.40 (10.50)	Medicated (*n* = 44)	0	3 T/11 mm	*p* < 0.05 (FWE)	MDD < HC: L/R SFG, L MFG
Scheuerecker et al. 2010	13 (3)	15 (5)	37.90 (10.10)	35.50 (10.90)	4.36 (5.96)	Drug naïve	0	3 T/8 mm	*p* < 0.001 (uncorr)	MDD > HC: R Cerebellum, R Rolandic operculum, R SFG, R Precuneus, L IFG, R Amygdala; MDD < HC: L IFG, L Inferior frontal operculum, L MTG, R Postcentral gyrus, L IPL, L STG, L Postcentral gyrus, L Rolandic operculum, R IOG, L IFG, ITG
Shad et al. 2012	22 (10)	22 (11)	15.00 (2.10)	16.00 (2.10)	NA	Medicated (*n* = 4)	2 anxiety disorder; 3 ADHD	1.5 T/8 mm	*p* < 0.05 (FWE)	MDD < HC: R IFG, L/R MFG, R SFG, L/R Caudate, R STG, L Thalamus, L/R Cerebellum
Sprengelmeyer et al. 2011	17 (9)	21 (12)	45.60 (12.30)	42.00 (12.90)	NA	Medicated (*n* = 17)	0	1.5 T/8 mm	*p* < 0.05 (Monte Carlo)	MDD < HC: L Insula, R PHG, L MTG, R SFG, L FFG, R Amygdala
Stratmann et al 2014	132 (76)	132 (74)	37.86 (11.87)	37.82 (11.42)	7.78 (8.79)	Medicated (*n* = 126)	41 anxiety disorder	3 T/8 mm	*p* < 0.05 (AlphaSim)	MDD < HC: R Insula, L SPL, L/R STG, L PHG
Straub et al. 2019	60 (48)	43 (38)	17.30 (3.44)	17.62 (3.85)	NA	Medicated (n = 20)	18 unspecified	3 T/6 mm	*p* < 0.05 (FWE)	MDD > HC: R dlPFC
Treadway et al. 2009	19 (10)	19 (10)	35.20 (10.50)	30.30 (8.60)	12.90 (13.70)	NA	7 anxiety disorder	3 T/12 mm	*p* < 0.05 (FWE)	–
Ueda et al. 2016	30 (13)	48 (13)	44.30 (13.00)	41.20 (11.40)	NA	NA	0	3 T/8 mm	*p* < 0.05 (FWE)	MDD < HC: STG
Wagner et al. 2011	30 (25)	30 (25)	37.55 (11.50)	35.10 (10.40)	5.59 (6.30)	NA	0	1.5 T/12 mm	*p* < 0.05 (FWE)	MDD < HC: L/R Nucleus caudatus, R IFG, R Subgenual cortex, L/R Hippocampus/amygdala, L OFC, L SFG
Wehry et al. 2015	14 (11)	41 (27)	14.00 (3.00)	13.00 (2.00)	NA	NA	5 ADHD; 1 disruptive behavior	4 T/8 mm	*p* < 0.001 (uncorr)	MDD > HC: R MFG, L Precuneus, R Thalamus, R Caudate
Yang et al. 2017	35 (35)	23 (23)	44.54 (11.14)	39.09 (14.35)	2.68 (3.75)	Drug naïve	NA	3 T/8 mm	*p* < 0.005 (AlphaSim)	MDD < HC: L/R MFG, L/R Insula, L/R Putamen, R Amygdala, R PHG, L Lingual gyrus, Cerebellum
Yüksel et al. 2018	37 (20)	54 (21)	37.90 (10.50)	35.90 (10.50)	NA	Medicated (*n* = 28)	13 OCD, somatoform or personality disorder	3 T/NA	*p* < 0.05 (FWE)	–
Zhao et al. 2017	37 (12)	41 (15)	26.70 (7.10)	27.10 (7.20)	2.00 (0.50)	Drug naïve	0	3 T/10 mm	*p* < 0.001 (FDR)	MDD > HC: R Temporal pole; MDD < HC: L/R OFC, L/R Putamen, L/R Thalamus, L/R MFG, L Cuneus
Zhou et al. 2018	144 (84)	111 (58)	28.17 (5.91)	27.63 (5.43)	NA	Drug naïve	0	3 T/8 mm	*p* < 0.05 (AlphaSim)	MDD < HC: R SOG
**Total Sample**	2575 (1636)	2866 (1644)	38.62 (14.19)^b^	37.42 (14.12)^b^	**Weighted t test**		sex: *t* = −2.47, *p* > 0.05		age: *t* = 3.51, *p* < 0.001	

*ACC* anterior cingulate cortex *AD* adjustment disorder with mixed disturbance of emotions and conduct, *ADHD* attention deficit hyperactivity disorder, *AG* agoraphobia, *AUD* alcohol use disorder, *corr* corrected, *dlPFC* dorsolateral prefrontal cortex, *dmPFC* dorsomedial prefrontal cortex, *FDR* false discovery rate, *FFG* fusiform gyrus, *FWE* family-wise error, *FWHM* full width at half maximum, *GRF* Gaussian random field, *HC* healthy controls, *IFG* inferior frontal gyrus, *IOG* inferior occipital gyrus, *IPL* inferior parietal lobule, *ITG* inferior temporal gyrus, *L* left hemisphere, *MCC* medial cingulate cortex, *MDD* major depressive disorder, *MFG* medial frontal gyrus, *MOG* middle occipital gyrus, *mPFC* medial prefrontal cortex, *MTG* middle temporal gyrus, NA not available, *OCD* obsessive-compulsive disorder, *OFC* orbitofrontal cortex, *PCC* posterior cingulate cortex, *PHG* parahippocampal gyrus, *R* right hemisphere, *SFG* superior frontal gyrus, *sgACC* subgenual anterior cingulate cortex, *SMA* supplementary motor area, *SOG* superior occipital gyrus, *SPL* superior parietal lobule, *STG* superior temporal gyrus, *uncorr* uncorrected, *VBM* voxel-based morphometry, *vmPFC* ventromedial prefrontal cortex.

^a^Studies that provided original whole-brain *t*-maps.

^b^Weighted averages. Note that the *p* values in the weighted t test have been corrected (Bonferroni) for multiple comparisons.

Comparing age and female ratio of the three patient groups with sample size-weighted one-way ANOVA revealed significant differences in mean age (*F*_2,75_ = 3 85, *p* < 0.05; *η*^2^ = 0.09) and a marginal significant difference in female ratio (*F*_2,75_ = 2.98, *p* = 0.057, *η*^2^ = 0.07). Post-hoc tests revealed that both mean age and female ratio of MDD patients were higher than those of the FAD (*ps* < 0.05). Age and sex were consequently included as covariates in the quantitative comparative meta-analyses.

Regarding comorbidity, 8.4% patients from GAD studies reported comorbidity with another anxiety disorder or MDD. 15.0% patients from FAD studies reported GAD or MDD comorbidity and only 5% MDD patients explicitly reported anxiety comorbidity. Although the comorbidity rates were not high in general, all comparative meta-analyses were recomputed with comorbidity percentages as covariates to exclude potential comorbidity effects (see Supplementary Table S2 an Fig. S1).

### Regional GMV alterations

#### GAD patients versus HC

**Fig. 2:**
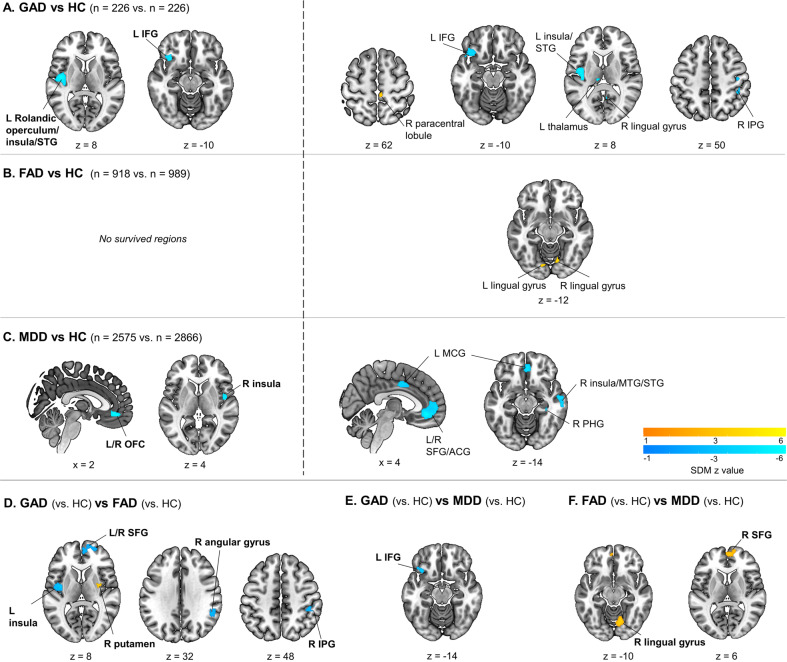
Common and disorder-specific gray matter volume alterations. Results of whole-brain meta-analysis of brain gray matter volume (GMV) among generalized anxiety disorder (GAD), fear-related anxiety disorder (FAD) and major depressive disorder (MDD). **A** Meta-analytic results for GAD relative to healthy controls (HC), **B** Meta-analytic results for FAD relative to HC, **C** Meta-analytic results for MDD relative to HC. The left panel shows brain regions significant at *p* < 0.05, TFCE corrected. The right panel shows brain regions significant at *p* < 0.0025, uncorrected. **D** Meta-analytic results for GAD (vs HC) in comparison to FAD (vs HC) covarying for age and sex, **E** Meta-analytic results for GAD (vs HC) in comparison to MDD (vs HC) covarying for age and sex, and **F** Meta-analytics results for FAD (vs HC) in comparison to MDD (vs HC) covarying for age and sex. Group comparisons are shown at *p* < 0.05, TFCE corrected. ACG anterior cingulate gyrus, IFG inferior frontal gyrus, IPG inferior parietal gyrus, L left, MCG median cingulate/paracingulate gyri, mPFC medial prefrontal cortex, MTG middle temporal gyrus, PHG parahippocampal gyrus, SFG superior frontal gyrus, STG superior temporal gyrus, OFC orbitofrontal cortex, R right.

Relative to HC (*n* = 226, from 9 studies), GAD (*n* = 226, from 9 studies) demonstrated robust GMV decreases in the left Rolandic operculum/insula/STG and left inferior frontal gyrus (IFG). No clusters of increased GMV were found (Table 4, Fig. 2A). When applying a more liberal threshold (*p* < 0.0025, uncorrected), decreased GMV was found in the left insula/Rolandic operculum/STG, left IFG, left thalamus, right lingual gyrus and right inferior parietal gyrus (IPG), while increased GMV was present in the right paracentral lobule (see Supplementary Table S1, Fig. 2A).

**Table 4:** Whole-brain meta-analysis results for VBM studies in GAD, FAD and MDD at threshold TFCE *p* < 0.05.

MNI coordinates	SDM Z	Voxels	Regions	BA	Egger’s bias	Egger’s p
**GAD** < **HC**						
−44, −8, 8	−3.951	507	L Rolandic operculum/insula/STG	48	0.45	0.806
−32, 24, −10	−3.973	110	L IFG, orbital part	38/47	−0.41	0.809
**FAD vs. HC**						
*None*						
**MDD** < **HC**						
2, 36, −10	−5.012	173	L/R SFG, medial orbital (OFC)	11	−0.54	0.300
46, −2, 4	−4.339	115	R insula	48	−0.37	0.405
**GAD** > **FAD**						
32, −6, 6	1.856	153	R putamen	48	0.50	0.654
**GAD** < **FAD**						
2, 54, 16	−2.102	480	L/R SFG, medial, dorsolateral (mPFC, dlPFC)	10	−0.31	0.618
−38,−10,10	−2.981	164	L insula/Rolandic operculum	48	−0.37	0.513
52,−48,32	−2.347	136	R angular gyrus	48	−0.35	0.530
44,−36,48	−2.267	41	R IPG	2	−0.34	0.552
**GAD** < **MDD**						
−36, 20, −14	−3.077	79	L IFG, orbital part	38	−0.38	0.302
**FAD** > **MDD**						
2,58,6	3.311	361	R SFG, medial (mPFC)	10	0.26	0.404
8,−72,−10	3.861	282	R lingual gyrus	18	0.20	0.495

*BA* Brodmann area, *dlPFC* dorsolateral prefrontal cortex, *FAD* fear-related anxiety disorder, *GAD* generalized anxiety disorder, *HC* healthy controls, *IFG* inferior frontal gyrus, *IPG* inferior parietal gyri, *L* left hemisphere, *MDD* major depressive disorder, *MNI* Montreal Neurological Institute, *mPFC* medial prefrontal cortex, *OFC* orbitofrontal cortex, *R* right hemisphere, *SDM* seed-based d mapping, *SFG* superior frontal gyrus, *STG* superior temporal gyrus, *TFCE* threshold-free cluster enhancement, *VBM* voxel-based morphometry.

Note: FAD included social anxiety disorder, panic disorder and specific phobia.

#### FAD patients versus HC

No significant differences between FAD (*n* = 918, from 23 studies) and HC (*n* = 989, from 23 studies) were found at TFCE-corrected *p* < 0.05 (Table 4). Subgroup meta-analyses in SAD (*n* = 444, from 10 studies) and PD (*n* = 370, from 11 studies) studies yielded no significant GMV alterations. The meta-analysis could not be conducted in SP (*n* = 104, from 2 studies) given the small number of original VBM studies. No AG studies were identified.

With a more liberal threshold, we identified increased GMV in the left and right lingual gyrus in FAD (Fig. 2B, Supplementary Table S1). Subgroup meta-analyses revealed increased GMV in a wide range of brain regions including the left and right lingual gyrus in SAD, and reduced GMV in the right insula, right STG and left temporal pole/STG in PD (Supplementary Table S1).

#### MDD patients versus HC

Patients with MDD (*n* = 2,575, from 46 studies) relative to HC (*n* = 2,866, from 46 studies) showed decreased GMV in the OFC and right insula. No clusters of increased GMV were found (Table 4, Fig. 2C). With a more liberal threshold, decreased GMV extended into the right insula/STG/Rolandic operculum, left and right medial OFC/anterior cingulate cortex (ACC), left and right median cingulate/paracingulate gyri (MCG), and right parahippocampal gyrus (PHG) ((Supplementary Table S1, Fig. 2C).

#### Comparison of GMV differences between GAD, FAD and MDD

Covarying for age and sex, there were significant GMV differences between patient groups at *p* < 0.05, TFCE corrected (Table 4). GAD was associated with disorder-specific decreased GMV relative to FAD in an mPFC cluster extending to the right dorsolateral PFC, left insula/Rolandic operculum, right angular gyrus and right IPG, whereas FAD had reduced GMV in the right putamen relative to GAD (Fig. 2D). Disorder-specific reduced GMV was observed in GAD relative to MDD in the left IFG (Fig. 2E). FAD, relative to MDD, had larger GMV in the right mPFC and lingual gyrus (Fig. 2F). In addition, including percentage of comorbidity as an additional covariate (together with age and sex) in the comparative meta-analyses yielded robust differences between GAD and FAD, as well as between FAD and MDD. The differences between GAD and MDD did not remain stable after additionally including comorbidity as a covariate, suggesting that the disorder-specific GMV abnormalities in the GAD versus MDD comparison might be partly influenced by comorbidity (see Supplementary Table S2 an Fig. S1).

#### GMV Conjunction Analyses

No clusters were identified in all conjunction analyses (i.e., GAD ∩ FAD ∩ MDD, as well as GAD ∩ FAD, GAD ∩ MDD and FAD ∩ MDD) at both *p* < 0.05, TFCE-corrected and *p* < 0.0025, uncorrected. Excluding studies with comorbid patients did not change the null findings.

### Meta-regression

With a threshold of TFCE-based FWE corrected *p* < 0.05 and voxel extent ≥10, meta-regressions suggested that the proportion of female patients within the GAD studies was negatively associated with smaller GMV in the left insula (117 voxels; MNI coordinates: −34, −16, 16; peak *Z* value: −2.874; Brodmann area 48), whereas no associations between GMV differences and any confounding variables were observed in FAD (i.e., age, sex, illness duration, comorbidity, medication) and MDD (i.e., age, sex, illness duration, comorbidity, medication, symptom severity). Of note, although some of the original studies were conducted in samples with comorbid disorders (see Tables 1–3), no effects of comorbidity were identified in the meta-regressions for FAD and MDD, suggesting no confounding effects of comorbidity on the main findings. Meta-regression on comorbidity could not be conducted in GAD given the insufficient number of studies (*n* < 9) reporting comorbid conditions.

### Analyses of heterogeneity and publication bias

Extraction of heterogeneity statistics *I*^2^ from the significant clusters indicated low heterogeneity (*I*^2^ < 50%). No significant publication bias was revealed by Egger’s test for any significant clusters in GAD, FAD and MDD (*ps* > 0.05, Table 4).

## Discussion

The present meta-analysis is the first to determine common and distinct neuroanatomical markers in anxiety-, fear-related AD and MDD in the context of DSM-5 nosology and psychopathological factor models. GAD exhibited decreased left insula volume relative to HC and FAD, as well as reduced volume of the adjacent ipsilateral IFG compared to HC and MDD. MDD exhibited decreased medial prefrontal volumes relative to HC and FAD yet not to GAD, while FAD exhibited increased lingual gyrus volume relative to HC and MDD and decreased putamen volume relative to GAD, yet no GMV alterations in prefrontal regions. No common structural abnormalities were found between the disorders (see Supplementary Fig. S3 for visualizing the distinct alterations as compared to controls). These findings provide first meta-analytic evidence demonstrating distinct neurobiological alterations in anxiety-, fear-related, and depressive disorders.

In line with a very recent meta-analysis reporting disorder-specific GMV alterations in AD vs MDD [28], the present study further demonstrated that the category of anxiety disorders can be neurobiologically separated along the anxiety vs fear dimension, thus providing a neurobiological foundation of the DSM-5 nosology and psychopathological factor models [3–6, 10]. Consistent with our hypothesis, GAD exhibited regional-specific GMV reductions in the left insula and IFG compared to HC at a strict threshold. The stricter thresholding together with the larger sample size (9 studies, 226 GAD patients) allowed us to identify a more specific GAD signature as compared to a previous meta-analysis in GAD that combined a small number of VBM studies (6 studies, 117 GAD patients) with a lenient threshold [24]. Our observation of decreased volume in insula and IFG in GAD aligns with findings from previous SBM studies reporting reduced cortical folding of the insula in GAD patients [125] as well as lower IFG cortical thickness in late-life GAD compared to age-matched HC [33]. The insula is implicated in interoceptive, salience and emotion processing [126]. Resting-state fMRI and lesion studies suggest that deficits in the Rolandic operculum/insula are associated with GAD as well as higher levels of anxiety and perceived stress which represent key symptoms of GAD [127, 128]. The IFG is critical for the implementation of top-down regulatory control [129]. Deficits in top-down control play an important role in GAD and patients with GAD have demonstrated reduced GMV [33], altered activation [130] and dysfunctional connectivity [55] of this region. Reduced GMV in the left insula and IFG may thus reflect deficient emotion regulation and inhibitory control of anxiety, stress and worrisome thoughts in GAD. Although the BNST plays a prominent role in animal and human models of anxiety [15, 45], we did not observe altered BNST volumes in GAD. This may suggest that pathological anxiety is not associated with volumetric alterations of the BNST or alternatively reflect general challenges to image the BNST using conventional MRI and the low sensitivity to detect volumetric BNST variations by means of VBM [131].

No significant GMV alterations were found in FAD, which might result from the clinical heterogeneity of FAD and is consistent with a recent meta-analysis indicating no shared GMV alterations between SAD and PD [29]. A more lenient threshold gave rise to increased GMV in the bilateral lingual gyrus in FAD, which partly confirmed our hypothesis in has been associated with symptom severity in SAD [36] and panic symptoms [132]. Altered functional activation in this region has been associated with abnormal sensory gating of emotional stimuli such as facial expressions in SAD [133] and PD [134]. Subgroup analyses for FAD further suggested that the increases in the lingual gyrus were mainly driven by SAD, which is in line with previous studies [36]. Lowering the statistical threshold additionally revealed greater volume of the left postcentral gyrus, STG/Rolandic operculum, and right superior parietal gyrus (SPG) in SAD and smaller GMV in the right insula and bilateral STG in PD, which resembles previous studies reporting altered cortical thickness in these regions in SAD (e.g., in the insula, parietal and postcentral regions) [135, 136], as well as in line with a previous meta-analysis in PD [29]. In contrast to our hypothesis, we did not observe structural alterations of the amygdala in FAD. The amygdala has been consistently involved in fear processing in animal studies [12, 137] and studies in healthy subjects [15, 16]. However, previous brain structural studies in FAD (e.g., SAD, PD, SP) yielded rather inconsistent findings with respect to amygdala volume alterations [23, 25, 29]. Future research using more sensitive approaches is needed to assess subtle variations in cortical and subcortical morphology in fear-related disorders [19]. The results of the FAD and the subgroup meta-analyses should be interpreted cautiously due to the less conservative statistical threshold. Nevertheless, our study extends previous single-disorder meta-analyses of GMV alterations in fear-related disorders and these findings may together reflect a neuroanatomical heterogeneity of the disorders that are commonly assigned to the fear dimension in the psychopathological factor model [4, 6].

The comparative meta-analysis between GAD and FAD revealed that GAD presented reduced GMV in the left insula/Rolandic operculum and mPFC, indicating a regional- and GAD-specific neuroanatomical marker. Notably, our study is the first meta-analysis demonstrating differentiated structural alterations in the insula in GAD relative to FAD, which aligns with a recent neurofunctional meta-analysis showing a GAD-specific hypoactivation of bilateral insula across cognitive and emotion domains in contrast to other AD [45]. Besides, GAD, as well as MDD, exhibited smaller GMV in the mPFC in comparison to FAD. Previous meta-analyses have identified altered volume in mPFC in GAD [24], MDD [30], and individuals with high neuroticism [138], a pathological meta-factor associated with GAD and MDD which share symptomatic (e.g., negative affect, worry) and genetic etiologies [8, 9]. fMRI studies have also shown common neurofunctional alterations in the mPFC and ACC in cognitive or emotional processing in both GAD and MDD [139–141]. Together, both structural and functional studies suggest shared medial prefrontal deficits in GAD and MDD. This is further exemplified in the present meta-analysis when comparing GAD and MDD with FAD. While previous studies also reported altered mPFC activity in SAD during social processing [142–144], previous meta-analytic results on GMV alterations in SAD and PD in this region remained inconsistent [23, 25, 29]. The mPFC is a key node in the anterior default mode network (DMN) and engaged in self-referential processing and emotion regulation including distress intolerance [145, 146]. Individuals with a distress-misery disorder history (e.g., GAD and MDD) are characterized by a reduced capacity to tolerate negative affect compared to individuals with fear-related disorders (e.g., SAD,PD and SP) [147]. Decreased mPFC volumes in GAD and MDD relative to FAD may represent a structural foundation for deficient distress tolerance in anxious-misery disorders. Notably, GAD also exhibited decreased GMV in the IFG relative to MDD. Previous fMRI studies reported disorder-specific hypoactivation in ventrolateral PFC which largely overlaps with IFG, during emotion regulation in GAD relative to PD [148] or MDD [140]. The decreased IFG volume may suggest deficient top-down control of exaggerated worry in GAD. However, the GMV difference between GAD and MDD did not remain robust after controlling for comorbidity, which may suggest that structural deficits in this region may be partly explained by complex comorbidity patterns between GAD and MDD.

The comparative meta-analysis between GAD and FAD additionally revealed lower putamen volume in FAD relative to GAD. This observation aligns with previous meta-analytic findings demonstrating decreased putamen GMV in FAD relative to HC and obsessive-compulsive disorder (OCD) [25, 29, 149], as well as with original studies reporting larger putamen volume in GAD compared to HC [54]. The putamen is part of the dorsal striatum and has been related to anxiety disorders and anxiety symptoms [150, 151]. For example, a previous VBM study indicated a positive relationship between intolerance of uncertainty, a psychological construct related to anxiety, and bilateral striatal volume, in particular the putamen [152]. In contrast, some evidence suggests a negative correlation between putamen volume and the severity of PD symptoms [67]. Previous studies on SAD reported inconsistent results with respect to striatal alterations. While some meta-analyses found decreased GMV of the putamen in SAD relative to HC [25, 29], other individual studies reported increased putamen volume in SAD [23, 153, 154]. The present study did not reveal significant GMV alterations in the striatum in the SAD subgroup analysis. This discrepancy may result from differences in the methodological approaches and the samples included. The present results are based on a whole-brain meta-analysis with a stringent threshold (FWE-corrected and *p* < 0.0025, uncorrected), whereas some previous studies employed a hypothesis-driven region of interest approach, or implemented a less conservative meta-analytic threshold (*p* < 0.005, uncorrected) and more lenient meta-analytic approaches. Future meta-analyses using more stringent approach might further clarify the structural changes of the striatum in AD. Prior fMRI studies revealed disorder-specific heightened putamen activation during incentive anticipation in SAD relative to HC and GAD [150]. Positron emission tomography (PET) studies have shown that SAD and PD are associated with compromised serotonergic (5-HT) neurotransmission in several brain areas including the putamen [155]. Decreasing the function of the 5-HT system has been reported to exacerbate psychological and physiological response to stressors in fear disorders (e.g., PD, SAD), but not in anxiety disorders (e.g., GAD) [156], which may relate to the GMV differences in the putamen between fear- versus anxiety-related disorders.

The results in MDD replicated previous meta-analyses reporting GMV reductions in the OFC/ventromedial PFC and right insula [28, 30]. Functional and structural deficits of the OFC have been repeatedly reported in MDD and are associated with the severity of rumination [157] and depressive symptom [158] in MDD, which may underlie cognitive, mood and social impairments. The insula has been found to play an important role in the pathophysiology of MDD [159, 160] and predict treatment response in MDD [161]. Our study specifically identified reduced GMV in the right mid-posterior insula which has been associated with interoception, somatosensory processes, and pain [162], consistent with previous studies showing that interoceptive abnormalities in MDD are associated with bilateral mid-posterior insula dysfunction [163]. Reduced GMV in mid-posterior insula has also been described in other mental disorders such as PTSD, schizophrenia and anorexia nervosa [160], suggesting that GMV reduction in this region may characterize mental disorders with dysfunctions in interoceptive processing. However, these previous studies and the current study on GAD revealed alterations in left mid-posterior insula, whereas our study points towards right mid-posterior insula deficits in MDD. Therefore, although the insula may be a potential biomarker for anxious-misery disorders, the dissociation of the left and right mid-posterior insula in GAD and MDD needs to be disentangled in future studies. In contrast to a previous meta-analysis we did not observe cerebellar volume reductions in MDD [28], which may be due to different inclusion criteria such that the previous study included treatment-resistant, remitted and first-episode MDD. Comparison with FAD indicated decreased volumes of the lingual gyrus in MDD, which is consistent with prior studies reporting reduced cortical thickness of the lingual gyrus in MDD relative to SAD [75].

The conjunction analyses did not yield common neuroanatomical alterations across the disorder groups, which was contrary to our hypothesis and previous transdiagnostic meta-analyses revealing transdiagnostic neural markers of psychopathology in the ACC/PFC and insula both structurally [27] and functionally [38]. However, these meta-analyses included both psychotic (e.g., schizophrenia) and non-psychotic disorders (e.g., substance use disorder, and OCD) and did not specifically aim at determining transdiagnostic alterations within internalizing disorders. In contrast to the transdiagnostic approach utilizing the pooled data, our combination of comparative and conjunctive meta-analyses allowed us to determine disorder-specific rather than unspecific neuroanatomical abnormalities in fear, anxiety, and depressive disorders. The discrepancy may additionally be due to the small number of GAD studies and the more stringent meta-analytic conjunction approach in combination with a conservative threshold in the present study. An additional analysis was conducted that pooled all studies to determine transdiagnostic brain alterations to increase comparability with previous transdiagnostic meta-analyses [27, 38]. This transdiagnostic meta-analytic approach revealed a transdiagnostic convergence in the right insula through pooling studies for all disorder groups (78 studies, 3797 patients, details see Supplementary Fig. S2), replicating prior transdiagnostic meta-analytical findings on reduced insula volume in combined psychotic (e.g., schizophrenia) and non-psychotic disorders (e.g., substance use disorder, and OCD) [27].

Several limitations should be considered. First, the number of studies in GAD was relatively small (9 studies). However, the number of studies met the recommended number of studies for applying SDM and the inclusion of one original map will have increased the sensitivity and power to detect robust GMV alterations [24]. Nevertheless, the statistical power for GAD may have been limited compared to FAD and MDD. Second, conceptually we only included GAD as a representative category of anxiety-related AD. Other anxiety-related disorders such as separation anxiety disorder were not included because there are no clear neural components for these categories. Third, the unbalanced number of studies in FAD subtypes and the two original maps included for SAD might bias the results towards specific subtypes of FAD such as SAD. Further, although we controlled the group differences in age and female ratio between MDD and FAD in the comparative meta-analyses and corresponding meta-regressions did not reveal evidence for an impact of these variables, we cannot fully exclude the potential of complex interaction effects between disorder-specific alterations and demographic differences between the disorder groups. Future comparative meta-analyses with an increasing number of original studies will be needed to allow sex- and age-matched subgroup meta-analyses to minimize the potential impact of demographic differences between the disorder groups [164]. Similarly, meta-analyses are limited with respect to controlling demographic differences between patients and healthy controls within disorder groups which are dependent on individual studies. More original studies with carefully matched control groups are required to better account for the potential effects of demographic variables such as age and sex. Finally, we employed a series of additional control analyses to explore the potential impact of comorbidity in the present meta-analysis including meta-regression, comparative meta-analyses with comorbidity percentage as a covariate and conjunction meta-analyses that excluded data from co-morbid samples. The primary results remained stable across different control analyses arguing against strong effects of comorbidity on the common and distinct GMV abnormalities. Nevertheless, we observed that the difference for GAD vs MDD in the IFG did not remain robust after including comorbidity as a covariate. Large scale projects capitalizing on individual studies such as the ENIGMA consortium are needed to facilitate the control for comorbidity effects on the individual level [19, 21].

Given the high prevalence and the detrimental personal and economic impact of internalizing disorders such as FAD, GAD and MDD [1], efficacious interventions are needed. However, a significant proportion of internalizing patients does not respond to the conventional psychotherapeutic or pharmacological intervention approaches [165–167]. To this end, neuromodulation strategies have gained increasing interest and the corresponding approaches may allow to directly target brain alterations in internalizing disorders. An increasing number of studies reported, for instance, a promising potential of repetitive transcranial magnetic stimulation (rTMS) in treating internalizing disorders [168–173]. While the conventional TMS stimulation approach is limited to the stimulation of cortical surface regions, other approaches such as real-time fMRI-informed neurofeedback may allow to modulate cortical and subcortical regions. Recent preclinical studies have demonstrated the potential of real-time fMRI-informed neurofeedback to modulate brain systems and circuits traditionally implicated in internalizing disorders [135, 174, 175] and suggest that the neurofeedback training success is associated with variations in regional GMV [176]. The efficacy of these interventions can benefit from the identification of robust and disorder-specific therapeutic targets from our comparative meta-analyses or other approaches (e.g., lesion network mapping [177]).

## Conclusions

Summarizing, this meta-analysis is the first to comprehensively investigate common and distinct GMV alterations in anxiety- and fear-related anxiety disorders, as well as depressive disorder, which bridges a gap between current psychiatric nosology (e.g., DSM-5, RDoC, psychopathological factor model) and neurobiological findings. In line with previous definitions, we found distinct neuroanatomical deficits underlying the pathophysiology of GAD, FAD and MDD with dissociated prefrontal and insula deficits in GAD and MDD characterized by anxious-misery, and striatum and threat detection deficits in FAD characterized by fear. The disorder-specific biomarkers could serve as therapeutic targets in future clinical practice.

## Supplementary information

Supplementary Methods and Results

## Data Availability

Coordinates and *t*-value files are available at https://osf.io/46uc2/. Unthresholded whole-brain maps are provided at https://neurovault.org/collections/11343/.
