# Supplementary material for: Pathological fear, anxiety and negative affect exhibit distinct neurostructural signatures: evidence from psychiatric neuroimaging meta-analysis
Source: Transl Psychiatry. 2022 Sep 23;12:405. doi: 10.1038/s41398-022-02157-9 (PMC9508096; doi:10.1038/s41398-022-02157-9)
Supplement: Supplementary file 1 — Supplementary Methods and Results [file 41398_2022_2157_MOESM1_ESM.docx]

**Supplement Methods**

**Literature search and inclusion**

Database search keywords for Generalized Anxiety Disorder (GAD) included: (“generalized anxiety disorder”, OR (“GAD”)) AND (“voxel-based morphometry”, OR (“VBM”), OR (“gray matter”)).

Database search keywords for Fear-related Anxiety Disorders (FAD) included: (“social anxiety disorder”, OR (“social phobia”), OR (“SAD”), OR (“specific phobia”), OR (“panic disorder”), OR (“Phobia”), OR (“agoraphobia”)) AND (“voxel-based morphometry”, OR (“VBM”), OR (“gray matter”)).

Database search keywords for Major Depressive Disorder (MDD) included: “Depression” AND (“voxel-based morphometry”, OR (“VBM”), OR (“gray matter”)). Broad search terms were used to prevent missing any relevant studies. Additionally, we inspected reference lists of relevant reviews and original studies to identify further eligible articles.

Inclusion criteria were (1) neuroimaging case control design studies comparing brain structure (i.e., gray matter volume/density) between a group of patients with validated primary diagnosis of GAD, FAD or MDD based on standard diagnostic systems (e.g., DSM, ICD) and healthy controls (HC) and (2) whole-brain VBM between-group comparisons (including null findings) reported. Studies were excluded if they (1) were non-empirical studies (e.g., review, meta-analysis, meeting abstract); (2) were not published in English; (3) participants > 65 years; (4) did not report direct comparison between a patient and healthy control (HC) group; (5) did not report whole-brain results (i.e., only ROI results); (6) did not provide sufficient data (e.g., peak coordinates, etc.) even after contacting the authors of the original study. In case the same HC reference group was employed for several diagnostic group comparisons (e.g., SAD vs HC, PD vs. HC) in a single study, the result for each pair of comparison was included in the meta-analysis.

**Meta-analysis**

*SDM procedures*

For each article group, raw statistical maps or coordinates from cluster peaks including their effective sizes (*t*-values, representing the GMV differences between patients and controls) and sample sizes were entered into the SDM-PSI to be preprocessed using a 20 mm full width half maximum (FWHM) anisotropic Gaussian kernel and 2 mm voxel size. In this step, SDM converts *t*-values into Hedge's g effect sizes and their variances using evaluated algorithms [1] in case raw maps for the original study are not available. SDM conducts multiple imputation to estimate the lower and upper bounds of possible effect sizes for each study using an anisotropic Gaussian kernel. Then in the mean analysis, SDM-PSI conducts unbiased maximum likelihood estimation (MLE) of effect sizes and associated standard errors by imputing the effect size maps of individual studies for each group based on MetaNSUE algorithms [2,3]. To prevent a single study or few studies from driving the results, SDM uses a leave one out jackknife procedure. The number of imputations for mean analysis was set to 50 by default. Finally, SDM-PSI allows a Freedman-Lane-based permutation test at the subject level to perform voxel-wise tests of statistical significance while controlling familywise error rate (FWER) using threshold-free cluster enhancement (TFCE) [4]. Details of these procedures are provided in the corresponding methodological publications [5,6] and the SDM-PSI reference manual (https://www. sdmproject.com/manual/).

**Meta-regression**

To control for potential confounding effects of demographic and clinical variables (e.g., age, sex, illness duration etc), meta-regression analyses were conducted for each group. Within the disorder categories some potential confounders could not be examined by means of meta-regression given that the corresponding data was only available for < 9 original studies (see recommendations in Radua, 2009[7]). In particular, the following variables could not be studied in GAD patients: illness duration, medication status, and severity of clinical symptoms. Moreover, the effect of severity of clinical symptoms could not be studied in FAD patients because the symptoms might vary conceptually between subcategories and were accordingly measured by different scales. Consequently, we have examined the effects of age and female ration in GAD, age, female ratio, illness duration, medication in FAD and MDD as well as symptom severity (Hamilton Depression Rating Scale, HDRS) in MDD.

**Controlling for comorbidity effects**

*Comparative meta-analyses*

To control for potential effects of comorbidity on the identified group differences in the comparative meta-analyses, the analyses were recomputed including comorbidity percentage as a covariate in addition to age and sex.

*Conjunction meta-analyses*

To control for the effects of comorbidity on the identification of shared GMV abnormalities between disorder groups, we excluded studies with comorbidity from the conjunction analyses. Given that there is currently no option to directly include a covariate in the conjunction meta-analyses, this approach allowed a comparably strict control of comorbidity. 7 GAD studies (192 patients), 13 FAD studies (364 patients) and 35 MDD studies (1606 patients) of non-comorbid patients were included in the conjunction analyses.

All results were thresholded at TFCE-based FWE corrected threshold *p* < 0.05 with a voxel extent ≥ 10.

**Transdiagnostic meta-analysis**

A transdiagnostic meta-analysis of VBM studies (78 studies, 3,719 patients) across GAD (9 studies, 226 patients), FAD (23 studies, 918 patients) and MDD (46 studies, 2,575 patients) was conducted and thresholded at TFCE-based FWE corrected *p* < 0.05.

**Supplement Results**

**Transdiagnostic meta-analysis**

Partly resembling the results of a previous transdiagnostic meta-analysis including schizophrenia, bipolar disorder, major depression, substance use disorder and a pooled group of anxiety disorders [8], we observed decreased right insular cortex volume in the overarching pooled meta-analysis (**Figure. S2**). However, the number of MDD studies was at least two times larger than the number of GAD or FAD studies and the imbalance may strongly bias the results in favor of the MDD-related alterations (see also results in the main text demonstrating reduced right mid-insula volumes in MDD but not FAD or GAD, **Figure 2A, B, C**), while the conjunction approach in our main analyses would only identify regions showing convergent alterations across the disorder categories.

| **Table S1.** Whole-brain meta-analysis results for VBM studies in GAD, FAD and MDD at *p* < 0.0025, uncorrected. | | | | | | | |
| --- | --- | --- | --- | --- | --- | --- | --- |
| **MNI coordinates** | **SDM Z-Score** | **P *value*** | **Voxels** | **Regions** | **BA** | **Egger’s bias** | **Egger’s p value** |
| **GAD > HC** |  |  |  |  |  |  |  |
| 4, -28, 62 | 3.411 | 0.00032 | 18 | R paracentral lobule | 4 | -0.37 | 0.832 |
| **GAD < HC** |  |  |  |  |  |  |  |
| -44, -8, 8 | -3.951 | 0.000039 | 411 | L insula, rolandic operculum, heschl gyrus, STG, supramarginal gyrus | 48 | 0.45 | 0.806 |
| -32, 24, -10 | -3.973 | 0.000035 | 247 | L IFG, temporal pole, insula | 38/47 | -0.41 | 0.809 |
| -8, -24, 12 | -3.694 | 0.00011 | 22 | L thalamus | -- | 0.26 | 0.878 |
| 6, -58, 10 | -3.117 | 0.00091 | 12 | R lingual gyrus | 17 | 0.77 | 0.697 |
| 44, -38, 50 | -3.177 | 0.00074 | 10 | R IPG | 2 | 0.28 | 0.873 |
| **FAD > HC** |  |  |  |  |  |  |  |
| 12, -70, -12 | 3.626 | 0.000144 | 26 | R lingual gyrus | 18 | -0.01 | 0.993 |
| -14, -80, -12 | 3.331 | 0.000433 | 20 | L lingual gyrus | 18 | 0.05 | 0.942 |
| **SAD > HC** |  |  |  |  |  |  |  |
| -56, -20, 22 | 3.514 | 0.000220 | 147 | L postcentral gyrus, STG | 48 | 0.26 | 0.797 |
| 48, -46, 34 | 3.476 | 0.000255 | 91 | R superior longitudinal fasciculus, angular gyrus | 39/40/48 | 0.60 | 0.602 |
| 26, -50, 58 | 3.484 | 0.000246 | 66 | R SPG | 5/7 | 0.11 | 0.909 |
| 24, 56, 12 | 3.641 | 0.000136 | 57 | R SFG, dorsolateral | 10 | 0.05 | 0.959 |
| -20, -32, 62 | 3.885 | 0.000051 | 35 | L postcentral/precentral gyrus | 3 | -0.08 | 0.933 |
| 56, -26, 48 | 3.342 | 0.000416 | 20 | R postcentral gyrus, supramarginal gyrus | 2/3 | 0.03 | 0.975 |
| -28, -42, -46 | 3.574 | 0.000176 | 19 | L cerebellum, | -- | -0.07 | 0.944 |
| -8, 52, 12 | 3.380 | 0.000363 | 16 | L SFG, medial | 10 | -0.31 | 0.74 |
| 14, -68, -10 | 3.145 | 0.000829 | 11 | R lingual gyrus | 18 | 0.15 | 0.874 |
| -12, -80, -12 | 3.265 | 0.000547 | 11 | L lingual gyrus | 18 | 0.56 | 0.587 |
| **PD < HC** |  |  |  |  |  |  |  |
| 38, 0, 8 | -3.784 | 0.000077 | 63 | R Insula | 48 | -0.79 | 0.488 |
| 48, -2, -10 | -3.200 | 0.000687 | 12 | R STG | 48 | -0.70 | 0.494 |
| -36, 10, -22 | -3.037 | 0.001195 | 12 | L temporal pole, STG | 38 | 0.09 | 0.928 |
| **MDD < HC** |  |  |  |  |  |  |  |
| 46, -2, 4 | -4.339 | 0.000007153 | 877 | R insula, MTG, STG, rolandic operculum | 48/21/22 | -0.37 | 0.405 |
| 2, 36, -10 | -5.012 | 0.000000298 | 722 | L/R SFG, medial orbital, L/R ACG | 11/10/32 | -0.54 | 0.300 |
| 10, 6, 40 | -3.709 | 0.000104129 | 118 | L/R MCG | 32/24 | -0.31 | 0.591 |
| 36, -30, -14 | -3.115 | 0.000919223 | 10 | R PHG | 20 | -0.59 | 0.371 |
| Abbreviations: ACG, anterior cingulate/paracingulate gyri; BA, Brodmann area; FAD, fear-related anxiety disorder; GAD, generalized anxiety disorder; HC, healthy controls; IFG, inferior frontal gyrus; IPG, inferior parietal gyri; ITG, inferior temporal gyrus; L, left hemisphere; MCG, median cingulate/paracingulate gyri; MDD, major depressive disorder; MNI, Montreal Neurological Institute; MTG, middle temporal gyrus; PHG, parahippocampal gyrus; R right hemisphere; SDM, seed-based d mapping; SFG, superior frontal gyrus; SPG, superior parietal gyri; STG, superior temporal gyrus; VBM, voxel-based morphometry. | | | | | | | |

| **Table S2.** Comparative meta-analysis for VBM studies covarying for age, sex and comorbidity at threshold TFCE |
| --- |

*p* < 0.05.

| **MNI coordinates** | **SDM Z** | **Voxels** | **Regions** | **BA** | **Egger’s bias** | **Egger’s p** |
| --- | --- | --- | --- | --- | --- | --- |
| **GAD > FAD** |  |  |  |  |  |  |
| 32, -8, -6 | 2.088 | 136 | R putamen | 48 | 0.48 | 0.649 |
| **GAD < FAD** |  |  |  |  |  |  |
| 46, -34, 48 | -2.662 | 1299 | R IPG | 2 | -0.38 | 0.499 |
| 24,56,12 | -2.469 | 1072 | L/R SFG, dorsolateral, medial | 10 | -0.35 | 0.525 |
| 14,-68,-10 | -2.671 | 74 | R lingual gyrus | 18 | -0.34 | 0.523 |
| -14,-78,-12 | -2.323 | 29 | L lingual gyrus | 18 | -0.35 | 0.550 |
| -40,-8,10 | -2.702 | 24 | L insula/Rolandic operculum | 48 | -0.36 | 0.530 |
| -6,44,-10 | -1.887 | 14 | L SFG, medial orbital | 10 | -0.28 | 0.618 |
| **GAD vs. MDD** |  |  |  |  |  |  |
| *None* | | | | | | |
| **FAD > MDD** |  |  |  |  |  |  |
| 12,-68,-10 | 3.261 | 618 | R lingual gyrus | 18 | 0.26 | 0.461 |
| 38,30,-4 | 2.923 | 62 | R IFG | 47 | 0.23 | 0.510 |
| 14,64,6 | 2.583 | 10 | R SFG | 10 | 0.21 | 0.550 |
| Abbreviations: BA, Brodmann area; FAD, fear-related anxiety disorder; GAD, generalized anxiety disorder; HC, healthy controls; IFG, inferior frontal gyrus; IPG, inferior parietal gyri; L, left hemisphere; MDD, major depressive disorder; MNI, Montreal Neurological Institute; R right hemisphere; SDM, seed-based d mapping; SFG, superior frontal gyrus; TFCE, threshold-free cluster enhancement; VBM, voxel-based morphometry.  Note: FAD included social anxiety disorder, panic disorder and specific phobia. | | | | | | |


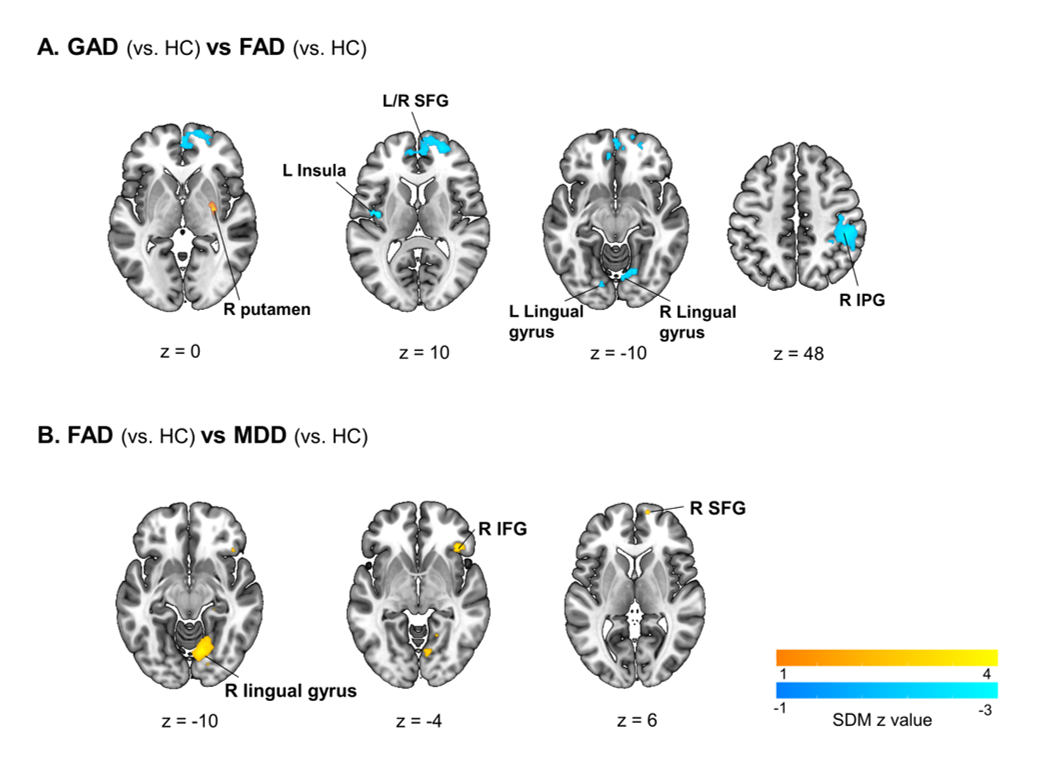
**Figure. S1**. Survived regions in the comparative meta-analyses covarying for age, sex and comorbidity between (A) GAD (vs HC) in comparison to FAD (vs HC), and (B) FAD (vs HC) in comparison to MDD (vs HC). No clusters were found between GAD (vs HC) and MDD (vs HC). Clusters were displayed at TFCE based FWE corrected *p* < 0.05.


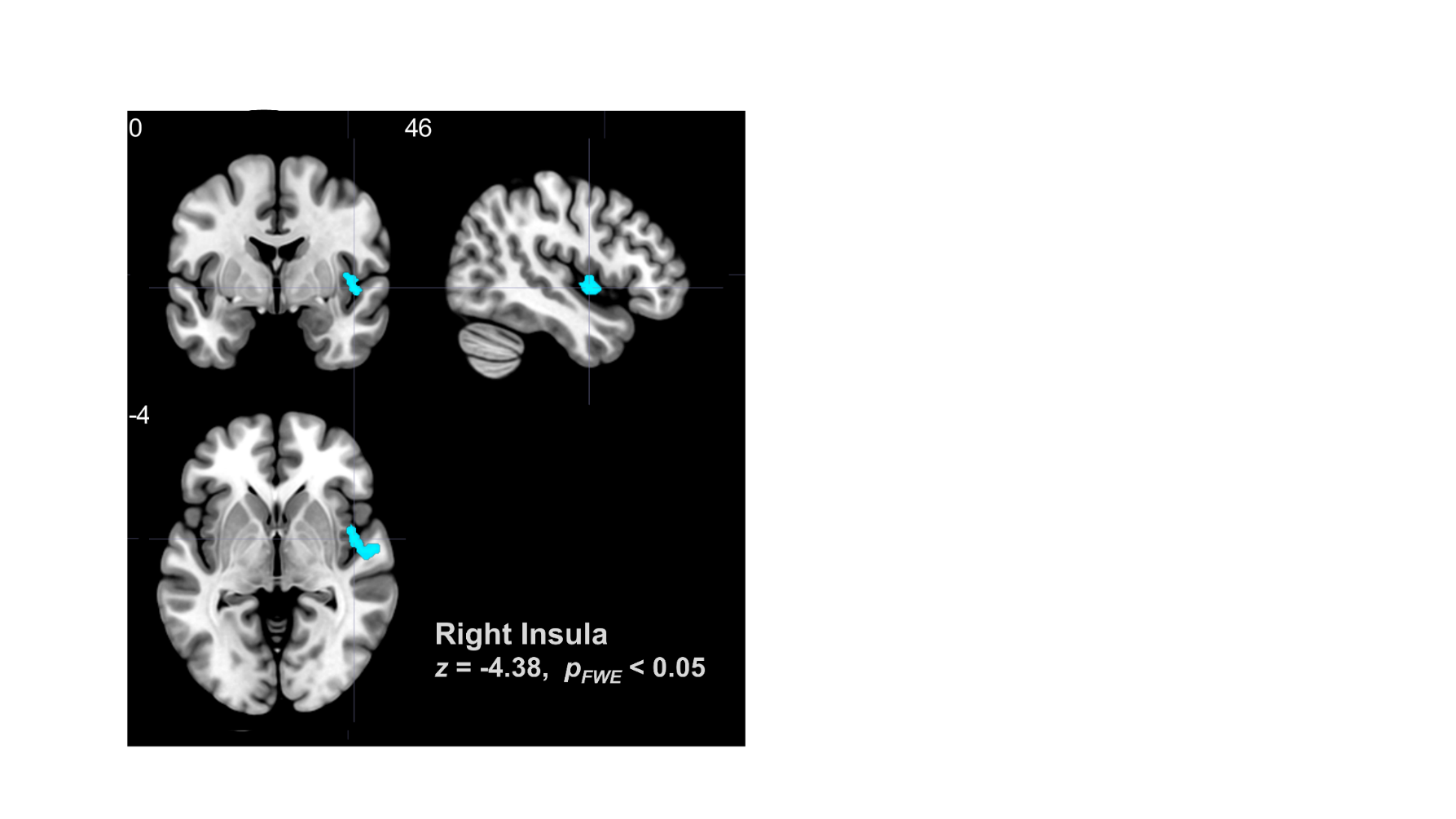


**Figure. S2**. Brain regions showing decreased GMV of patients vs healthy controls for studies pooled across GAD, FAD and MDD (78 studies, 3,719 patients). Clusters were displayed at TFCE based FWE corrected *p* < 0.05.

**
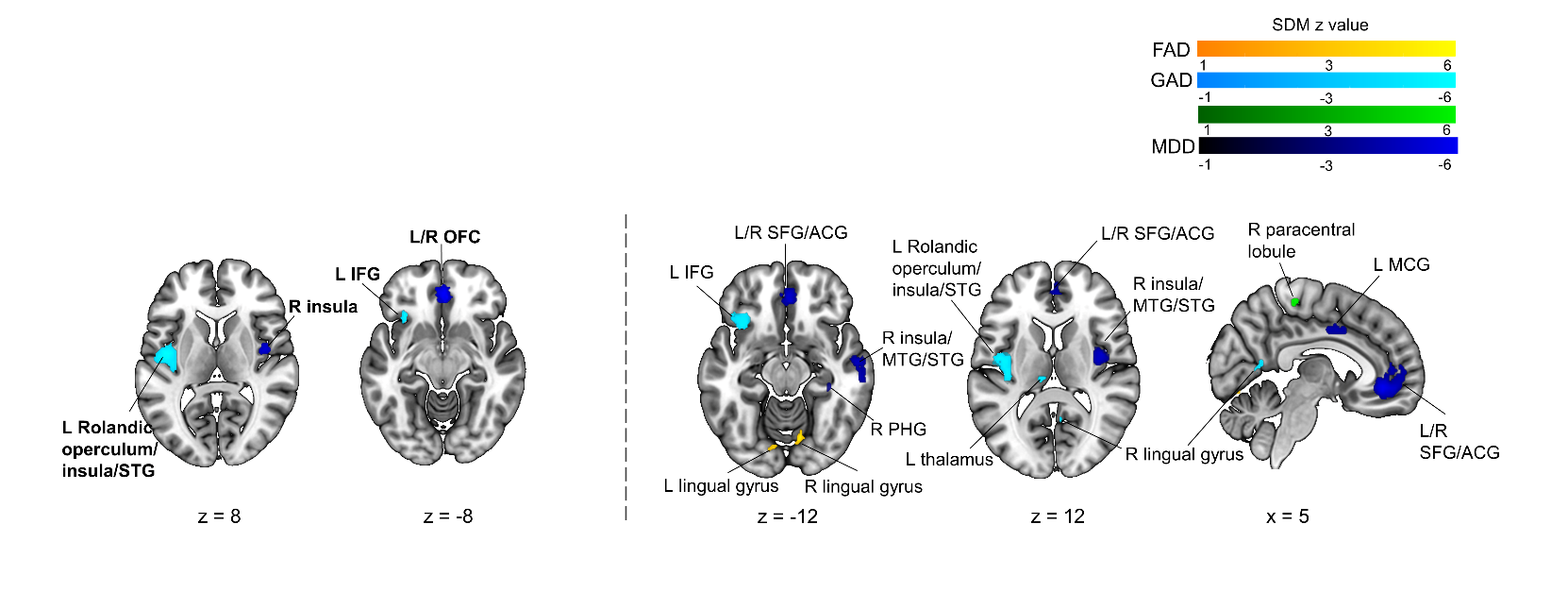
Figure. S3**. Brain regions showing GMV changes of GAD, FAD, MDD vs healthy controls in one single overlay. Both GAD and MDD showed decreased GMV in prefrontal and insula cortex, whereas FAD showed an increase in lingual gyrus. Left panel shows brain regions significant at *p* < 0.05, TFCE corrected. Right panels show brain regions significant at *p* < 0.0025, uncorrected.

**Supplement References**

1 Radua J, Mataix-Cols D, Phillips ML, *et al.* A new meta-analytic method for neuroimaging studies that combines reported peak coordinates and statistical parametric maps. *Eur Psychiatry* 2012;**27**:605–11. doi:10.1016/j.eurpsy.2011.04.001

2 Radua J, Schmidt A, Borgwardt S, *et al.* Ventral Striatal Activation During Reward Processing in Psychosis: A Neurofunctional Meta-Analysis. *JAMA Psychiatry* 2015;**72**:1243–51. doi:10.1001/JAMAPSYCHIATRY.2015.2196

3 Albajes-Eizagirre A, Radua J. What do results from coordinate-based meta-analyses tell us? *Neuroimage* 2018;**176**:550–3. doi:10.1016/J.NEUROIMAGE.2018.04.065

4 Smith SM, Nichols TE. Threshold-free cluster enhancement: Addressing problems of smoothing, threshold dependence and localisation in cluster inference. *Neuroimage* 2009;**44**:83–98. doi:10.1016/J.NEUROIMAGE.2008.03.061

5 Albajes-Eizagirre A, Solanes A, Vieta E, *et al.* Voxel-based meta-analysis via permutation of subject images (PSI): Theory and implementation for SDM. *Neuroimage* 2019;**186**:174–84. doi:10.1016/j.neuroimage.2018.10.077

6 Albajes-Eizagirre A, Solanes A, Fullana MA, *et al.* Meta-analysis of voxel-based neuroimaging studies using seed-based d mapping with permutation of subject images (Sdm-psi). *J Vis Exp* 2019;**2019**. doi:10.3791/59841

7 Radua J, Mataix-Cols D. Voxel-wise meta-analysis of grey matter changes in obsessive-compulsive disorder. *Br J Psychiatry* 2009;**195**:393–402. doi:10.1192/bjp.bp.108.055046

8 Goodkind M, Eickhoff SB, Oathes DJ, *et al.* Identification of a common neurobiological substrate for mental Illness. *JAMA Psychiatry* 2015;**72**:305–15. doi:10.1001/jamapsychiatry.2014.2206
